# Standard Deviation Effect of Average Structure Descriptor on Grain Boundary Energy Prediction

**DOI:** 10.3390/ma16031197

**Published:** 2023-01-31

**Authors:** Ruoqi Dang, Wenshan Yu

**Affiliations:** State Key Laboratory for Strength and Vibration of Mechanical Structures, Shaanxi Engineering Laboratory for Vibration Control of Aerospace Structures, School of Aerospace Engineering, Xi’an Jiaotong University, Xi’an 710049, China

**Keywords:** grain boundary, descriptor, pair distribution function, grain boundary energy, machine learning method

## Abstract

The structural complexities of grain boundaries (GBs) result in their complicated property contributions to polycrystalline metals and alloys. In this study, we propose a GB structure descriptor by linearly combining the average two-point correlation function (PCF) and standard deviation of PCF via a weight parameter, to reveal the standard deviation effect of PCF on energy predictions of Cu, Al and Ni asymmetric tilt GBs (i.e., Σ3, Σ5, Σ9, Σ11, Σ13 and Σ17), using two machine learning (ML) methods; i.e., principal component analysis (PCA)-based linear regression and recurrent neural networks (RNN). It is found that the proposed structure descriptor is capable of improving GB energy prediction for both ML methods. This suggests the discriminatory power of average PCF for different GBs is lifted since the proposed descriptor contains the data dispersion information. Meanwhile, we also show that GB atom selection methods by which PCF is evaluated also affect predictions.

## 1. Introduction

Grain boundaries (GBs) are one of the most commonly seen planar defects in polycrystalline metals and alloys. Due to local atomic distortions and inconsistent atomic arrangement, GBs play important roles in determining the mechanical, thermal and electric, etc., properties of materials [1,2]. For example, GBs may act as the dislocation and point defect sources or sinkers, and they may block the dislocation motion and absorb them; thus the strength and ductility of materials can be greatly changed [3]. For an idealized GB, from the geometrical point of view, it can be completely governed by five parameters, usually represented by misorientation and a normal GB plane [4]. Unfortunately, the structures, as well as the properties of the GB, are hard to completely determine. This is simply because a GB may have numerous states due to the point defect absorptions and emissions. It means the structures of a given GB may no longer be unique for a given energy [5,6,7,8,9,10,11]. Thus, the connection between structure and property of a GB, such as energy, volume and mechanical behavior, etc., are usually built via atomistic simulations by using molecular dynamics (MD) and density functional theory (DFT) methods [12,13], which is also of great significance for the macroscopic modeling of material behavior [14,15]. Technically speaking, it is possible to do so using MD and DFT, but also needs a heavy workload if such connections for a large number of GBs are expected. 

The ML method has been applied in many research fields [16,17,18,19,20], and provides an efficient technique by which to link the structure-property of a GB, particularly to extract correlations from high-dimensional datasets [21,22,23,24,25], and has been successfully applied in predicting GB energies [21,23,24,25,26,27], point defect segregation energies [28,29], GB structures [30] and damages and deformations in GB [31,32]. Usually, an appropriate ML method is employed according to the datasets and the expected correlations. Regardless of these, a problem is how to mathematically describe the GB structure, which should contain the essential characteristics of GB structures. One of the representative examples is structure units (SUs) [33,34,35,36], which are usually used to describe GB structures, but are incapable of doing so for general GBs or GBs driven out of equilibrate states [11]. Furthermore, some studies have also focused on developing structural matrices [12,13,37]. We could indeed gain a unique insight from these studies. However, these descriptions cannot be readily applied in ML. 

So far, some descriptors related to GB structures, which can be well used in ML so that the atomic structure-property relationships can be constructed, have been developed. Generally, these descriptors can be divided into two categories; i.e., local atom environment descriptor and descriptors for atom connectivity [21,22,38,39]. The former mostly considers the local atom environment, such as the atomic neighboring arrangement. One of the representative examples, developed by Banadaki and Patala [38,39], describes the local GB structure by using some polyhedral units in a way similar to SUs. In principle, this method can be used to represent any general GB. However, GB structures are usually not at a equilibrate state or subjected to deformation perturbations due to vacancies or self-interstitial diffusions, absorptions and emissions. It means this technique itself suffers from limitations. Banadaki et al. proposed the point-pattern matching algorithm to enhance the power of PU for describing GBs, particularly with local distortions [38]. Another example is the smooth overlap of atomic positions (SOAP) descriptor, which is essentially a combination of radial and spherical spectral bases, including spherical harmonics [22]. For the atom connectivity descriptors, they essentially correlate the positions of atoms in GB and in the vicinity of GB. Such descriptors usually vary smoothly upon the perturbations of atom positions [22]. An example is the pair correlation function (PCF) method due to Gomberg et al.’s study [21]. It was shown that the two-point correlation functions can reliably predict GB energies. Based on these descriptors, a variety of GB properties, such as energy, mobility and mechanical behavior, etc., can be successfully predicted by using ML methods. 

Using ML methods to predict GB properties, a better quantitative prediction usually requires that a descriptor should carry the information of the GB structure as much as possible [38]. An extreme case is to consider all atoms composed of the GB structure by evaluating the neighboring atom distribution for each GB atom [22]. This not only drastically increases the dimension of data, but may also lead to data dimension inconsistency between GBs. To avoid such a dilemma, a straightforward method is to further compute the average quantity [21,38]. This way, the descriptor can be seen as an average structure representation (ASR) [22]. From the statistics, ASR does not contain the information of data scatter, such as the standard deviation. Moreover, it is still unclear how standard deviation affects the prediction. 

In this study, we establish 464 asymmetric GB models for Cu, Al and Ni metals and relax all GB models using MD. We discuss the prediction of GB energies by comparing two ML methods, i.e., principal component analysis (PCA) -based linear regression [40] and recurrent neural networks (RNN) [41,42], in an effort to answer the following questions:

Based on two-point correlation functions, i.e., the PCF method proposed by Gomberg et al. [21], we introduce a new GB structure descriptor by linearly combining PCF and its standard distribution PCF_std_ via a parameter. How does such a descriptor affect the prediction? 

GB atoms can be selected from GB using common neighbor analysis (CNA) [43] and centro-symmetric parameter (CSP) methods [44]. It can be imaged that a different number of GB atoms could be selected for two methods when setting different critical values. Will this affect the prediction? 

Comparing two ML methods, what happens to the prediction when considering the GB atom selection method and the GB structure descriptor? Can we predict GB energy without clearly distinguishing the tilt axis of the GB?

The content of this paper is organized as follows. Firstly, we introduce the GB models and the establishment of the GB structure descriptor. Secondly, the PCA-based linear regression of energies of Cu, Al and Ni GBs according to the tilt axis of GB considering full data and partition data is discussed. Thirdly, the effect of standard deviation of PCF on the RNN-based prediction of GB energies is discussed. Finally, the prediction comparisons between the two ML methods and conclusions of this study are made. 

## 2. Methodology and GB Structure Descriptor

We consider a total number of 464 asymmetric tilt GBs (ATGBs) with misorietations Σ3, Σ5, Σ9, Σ11, Σ13 and Σ17 as the dataset for the subsequent GB energy prediction study. Each GB model is a bi-crystal composed of two grains with specified orientations. To construct it with periodic boundary conditions (PBCs) applicable, crystalline orientations of two grains are needed. Usually, a given GB misorientation Σ can be defined by the overlapped lattices of two crystals with one of them rotated around a specified axis ρ with a certain angle θ. Namely, Σ is equivalent to (ρ, θ), which can also be represented by a rotation matrix **R**_Σ_. Take Σ = 3 as an example, (ρ, θ) related to Σ3 equals ([110], 70.53°) [33], and the corresponding unit cell of Σ3 coincidence site lattice (CSL) is spanned by [1$\overset{-}{1}$0], [111] and [11$\overset{-}{2}$]. Then, the normal GB **m** of a series of Σ3 ATGBs expressed in one grain are linear combinations of [111] and [11$\overset{-}{2}$], i.e., *i* [111] + *j* [11$\overset{-}{2}$] for different integers *i* and *j*. GBs normally expressed in another grain can be obtained by **R**_Σ3_.**m**. The angle between **m** and [111] defines the inclination angle, denoted as *ϕ*. Using **m** and **R**_Σ3_.**m**, along with the tilt axis [1$\overset{-}{1}$0], the crystal orientations of two grains of all Σ3 ATGBs can be defined. By following this approach, asymmetric GBs of all other misorientations can be readily created. 

For Σ3, Σ5, Σ9, Σ11, Σ13 and Σ17, lattice symmetry requires the inclination angle *ϕ* varying from 0° to 90° for Σ3, Σ9 and Σ11, with *ϕ* varying from 0° to 45° for Σ5, Σ13 and Σ17, respectively. PBCs are imposed within the GB plane for all bicrystal models. Two grains (i.e., Grains A and B) terminate with free surfaces in the direction perpendicular to the GB plane by setting two 10Å thick vacuum spaces on the top and bottom ends of the bicrystal model, as schematically shown in Figure S1 in Supplementary Materials. This allows us to release the stress possibly produced in the z direction during the GB structure optimization. Embedded atom method (EAM) potentials [45,46] are used to model the atomic interactions in Al [47], Cu [48] and Ni [47]. We relax all 464 ATGBs via the conjugate gradient (CG) method using LAMMPS [49] and compute the average energies of all GBs. Tables S1 and S2 in Supplementary Materials list the variations in atom numbers and energies for Σ3, Σ5, Σ9, Σ11, Σ13 and Σ17 GB models of each metal. Atomic structures are visualized using Ovito [50].

With all GBs relaxed, we are in a position to introduce the descriptor by which the GB structure and structure differences between GBs can be described and distinguished. Herein, we employ the pair correlation function (PCF) method proposed Gomberg et al. [21] as a GB structure descriptor. In doing so, a primary concern is how many atoms in GB should be considered when evaluating the average PCF (PCF_mean_(r)). In other words, an appropriate method for selecting GB atoms should include a certain number of atoms in the vicinity of the GB carrying structure information, but exclude other atoms. Herein, we consider common neighbor analysis (CNA) [43] and centro-symmetric parameter (CSP) methods [44]. For the CSP method, two CSP critical values are considered (i.e., CSP > 0.1 and 0.5). As exemplified in Figure S2 in Supplementary Material, the three methods identify a different number of GB atoms. Such effects on GB energy predictions will be discussed in the following section.

According to the approach of Gomberg et al. [21], the PCF of a given GB is computed by averaging the radial distribution function $g_{a}\left(r\right)$ of all *N*_GB_ GB atoms selected out of the GB using CNA, CSP_0.1_ and CSP_0.5_, which can be expressed as

(1) $${PCF}_{mean}\left(r\right)=\frac{1}{N_{GB}}\sum_{\alpha \in GB}g_{a}\left(r\right)$$

where the radial distribution function $g_{a}\left(r\right)$ of a GB atom can be calculated by considering all of its *N_in_* neighboring atoms within a specified cut-off radius for three metals.

(2) $$g_{\alpha }\left(r\right)=\sum_{k=1}^{N_{in}}\frac{K^{e}(r-\left\|R_{\alpha }^{k}\right\|,h^{e})}{4\pi r^{2}n_{0}}$$

where a kernel function *K^e^* with bandwidth *h^e^* is used to smoothen the radial distribution function. *n*_0_ is the atom density in the FCC lattice and $\left\|R_{\alpha }^{k}\right\|$ is the distance between atom *a* and its *k*th neighboring atom. The parameters needed in Equation (2) are listed in Table S3 in Supplementary Material.

**Figure 1:**
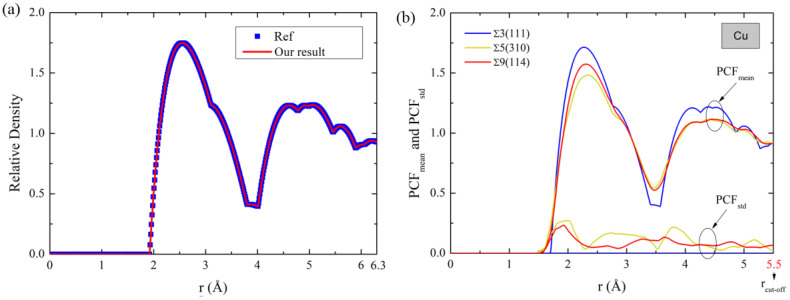

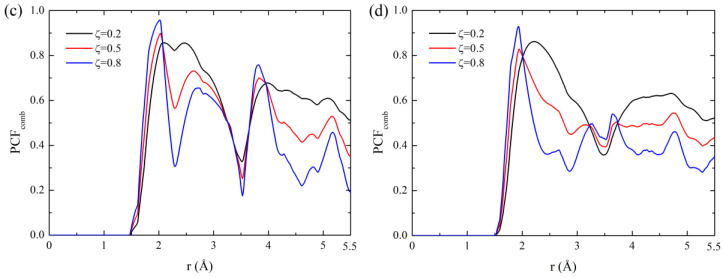
(**a**) PCF of Al single crystal compared with the results taken from [16], (**b**) PCF_mean_ and PCF_std_(r) of Cu GBs ∑5(310), ∑9(114) and ∑3(111). PCF_comb_(r) of Cu GBs (**c**) ∑5(310) and (**d**) ∑9(114) for ζ = 0.2, 0.5 and 0.8. Results of figure (**b**) for Al and Ni are shown in Figure S3 in Supplementary Material.

Figure 1a compares PCF_mean_ of the Al single crystal with results taken from [21]. Good agreement validates our algorithm for computing PCF_mean_. In fact, PCF_mean_ is an averaged radial distribution function (RDF) curve of each GB atom, by which the PCF data fluctuations of different GB atoms cannot be well considered, as evidenced by the variation in standard deviation of PCF (PCF_std_(r)) for three GBs in Cu in Figure 1b. In order to incorporate the data fluctuation into the averaged PCF, we further propose a PCF_comb_ by combining PCF_mean_(r) and PCF_std_(r) as

(3) $${PCF}_{comb}\left(r\right)=\left(1-\zeta \right)\frac{{PCF}_{meam}\left(r\right)}{\max\left({PCF}_{meam}\left(r\right)\right)}+\zeta \frac{{PCF}_{std}\left(r\right)}{\max\left({PCF}_{std}\left(r\right)\right)}$$

where parameter ζ is introduced to weigh the portions of PCF_mean_(r) and PCF_std_(r) in PCF_comb_. PCF_comb_ is reduced to PCF by letting ζ=0. As an example, Figure 1c,d shows the PCF_comb_(r) of ∑5(310) and ∑9(114) Cu GBs for three values of ζ. Clearly, the variation trends of PCF_comb_(r) changes as ζ varies. In the following, how the variation of ζ influences the prediction will be discussed. The PCF_comb_ curve of each GB is further represented as 512 discrete points, serving as the input data for the ML methods.

In the following, we adopt two ML methods to predict GB energies, i.e., principal component analysis (PCA)-based linear regression [40] and recurrent neural networks (RNN) [41,42]. PCA is usually implemented in two steps, a dimensionality-reduction of data and regression based on the principle component, which are essentially the eigenvalues of the covariance matrix of raw data. Therefore, the regression of PCA is achieved only using a few principle components. The principle components for regression are selected by considering the explained variance percentage of each principle component. However, RNNs do not require dimensionality reduction. The training and prediction are performed by using raw data. To quantitatively compare the predictions, mean absolute error (MAE) and mean relative error (MRE) are assessed via

(4) $$MAE=\frac{1}{n}\sum_{i=1}^{n}\left|\gamma_{i}^{Pred}-\gamma_{i}^{MD}\right|$$

(5) $$MAE=\frac{1}{n}\sum_{i=1}^{n}\left|\gamma_{i}^{Pred}-\gamma_{i}^{MD}\right|/\gamma_{i}^{MD}$$

where $\gamma_{i}^{Pred}$ and $\gamma_{i}^{MD}$ are GB energies predicted by ML methods and computed via MD.

## 3. PCA-Based Prediction

To implement PCA, we need to determine which principle components will be used in the regression. To do so, we analyze the explained variance percentage of the first ten principle components for Cu, as shown in Figure S4 in Supplementary Materials. It turns out that the explained variation of the first PC is up to 93%, while those of the other nine PCs are lower than 3%. This suggests that only the first few PCs accounting for higher explained variance percentages retain most of the original data, while the rest only keep a small amount of the data. It is therefore unnecessary to consider many PCs in the subsequent GB energy regression. Because of this, only the first three PCs, e.g., PC_1_, PC_2_ and PC_3_, are used in the regression. With the multiple linear regression method, GB energy can be written as

(6) $$\gamma_{GB}=a.PC_{1}+b.PC_{2}+c.PC_{3}+d$$

where a, b, c and d are fitting parameters. PC_1_, PC_2_ and PC_3_ are obtained from data training, which is dependent on the dataset. The dataset in this study consists of GBs with <100> and <110> tilts axes. Thus, there are two possible ways to obtain PCs by reducing the dimensionality of data when considering all data together and two data subsets corresponding to <100> and <110> tilt axes, denoted as *full data* and *partitioned data* methods, respectively. Thus, two sorts of PCs PC_i_ through training different datasets can be obtained.

The PCs obtained from data training are actually a representation of data in a lower dimensional space. Although these PCs retain most of the original data, it is still challenging to impart each PC with possible physical interpretability. By following the approach by Gomberg et al. [14], it is possible to correlate each PC to a geometrical parameter of GB by interpolating each PC as a function of the geometrical parameter. Herein, such a geometrical parameter is considered to be inclination angle *ϕ* related to asymmetric GBs for a specified Σ. In this study, PC_1_, PC_2_ and PC_3_ are assumed to be cubic polynomial interpolation functions of *ϕ*.

(7) $$PC_{i}\left(\varphi \right)=A_{i}\varphi^{3}+B_{i}\varphi^{2}+C_{i}\varphi +D_{i}$$

where A_i_, B_i_, C_i_ and D_i_ are fitting parameters. Such cubic polynomial interpolation can well characterize the variation of calculated PC_i_ vs *ϕ* for most GBs and PCs. From the above analysis, there are four ways of predicting GB energies in terms of different approaches of obtaining PCs, denoted as *full data*, *full data-fitting*, *partitioned data* and *partitioned data-fitting* methods, respectively. For the partitioned data, Equation (4) corresponds to three metals, shown in Equations (S2)–(S8) in Supplementary Material. The parameters in Equation (5) are listed in Tables S4–S6 in Supplementary Material.

**Figure 2:**
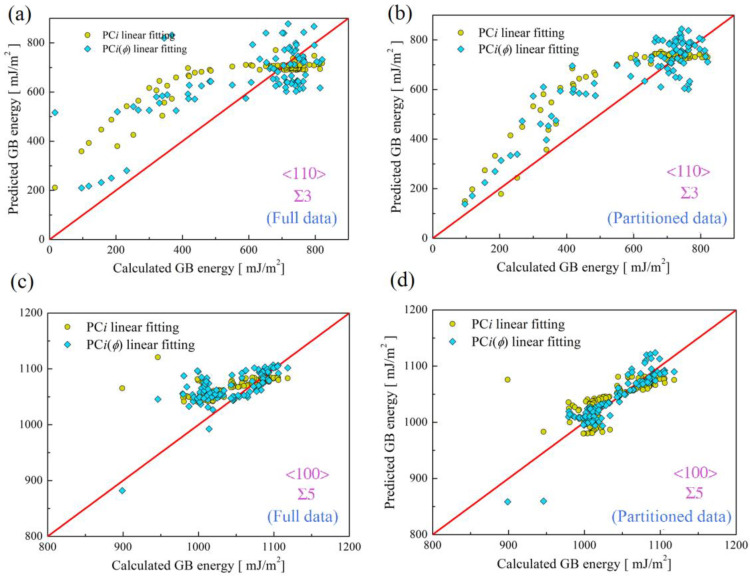
Energy predictions for (**a**,**b**) Σ3 and (**c**,**d**) Σ5 asymmetric copper GBs. Dimensionality reductions are performed based on full data (Figures (**a**,**c**)) and partitioned data (Figures (**b**,**d**)). Note that this figure exemplifies the predictions using PCF_comb_ computed for CNA-based GB atom selection and ζ = 0.5. Results of Σ9, Σ11, Σ13 and Σ17 GBs based on full data and partitioned data are shown in Figures S5–S8 in Supplementary Material.

For comparison, Figure 2 exemplifies energy predictions of Σ3 and Σ5 asymmetric copper GBs related to <110> and <100> tilt axes. In order to assess the prediction improvement due to the data partition, MAEs of all predictions are calculated, as shown in Figure 2. For Σ3 GBs, MAEs for PCi- and PCi(ϕ)-based linear fittings using full data are 64.57 mJ/m^2^ and 113.77 mJ/m^2^, but those for partitioned data are 45.49 mJ/m^2^ and 87.42 mJ/m^2^. For Σ5 GBs, MAEs for PCi- and PCi(ϕ)-based linear fittings using full data are 25.58 mJ/m^2^ and 29.62 mJ/m^2^, but those for partitioned data are 4.29 mJ/m^2^ and 10.66 mJ/m^2^. From further inspection of the variation of MAEs due to the data partition, MAEs for PCi- and PCi(ϕ)-based linear fittings are reduced by ~30% and ~23%, respectively, but, for Σ3 GBs, they are up to ~83% and ~64%. This suggests that a better prediction can be achieved by separately considering <110> and <100> GB datasets, which is particularly more prominent for <100> GBs. From the MAEs results of PCi and PCi(ϕ) linear fittings for Σ3 and Σ5 GBs, PCi(ϕ) linear fittings indeed lead to a larger MAE than PCi linear fittings, which is understandable since PCi(ϕ) is approximately obtained from cubic interpolation. Nevertheless, these results show that PCi is capable of being correlated with inclination angle ϕ.

As previously mentioned, $\gamma_{i}^{Pred}$ should be dependent on ζ, therefore, both MAE and MRE are functions of ζ. As an example, Table 1 shows MAEs and MREs of all Σs for ζ = 0.5. Comparing the MAE and MRE predictions for <100> and <110> GBs, both MAE and MRE are lower for <100> GBs, which further demonstrates that better predictions can be obtained for <100> GBs. In fact, this can be explained by considering the structure differences of <110> and <100> GBs. It is known that SUs for <100> GBs are composed of some [100] dislocations [2,51,52]. This brings simpler and mutually similar structures to <100> GBs. However, <110> GBs are composed of SUs much more complicated than those of <100> GBs [2,33,53,54,55]. Therefore, the structures of two <100> GBs may be quite different from each other. Thus, predictions for <100> GBs are better than those for their <110> counterparts, as also evidenced by the results of Al and Ni (see Figures S11, S12, S16 and S17 in Supplementary Material).

**Figure 3:**
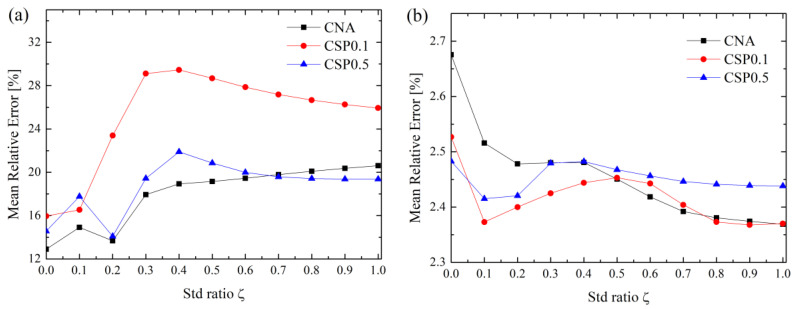
MRE of Cu GBs for three methods of selecting GB atoms (**a**) <110> and (**b**) <100> tilt axis. Results of Al and Ni are shown in Figures S15 and S20 in Supplementary Material.

In order to compare the effects of the GB atom selection method (i.e., CNA, CSP = 0.1 and CSP = 0.5) on the prediction, Figure 3a,b exemplify the MRE of <110> and <100> Cu GBs vs. ζ. From Figure 3, with increasing ζ, the MRE of <110> GBs keep increasing; however, that of <110> GBs keep decreasing. Finally, MREs of both <110> and <100> GBs reach plateaus. Further inspection of Figure 3 reveals that the minimum values of MRE for <110> and <100> GBs for CAN and CSP0.1 methods correspond to ζ = 0.0 and 1.0, respectively. For the CSP0.5 method, the minimum values of MRE for <110> and <100> GBs are ζ = 0.2 and 0.1. Moreover, considering CAN, CSP0.1 and CSP0.5 alone, MREs also differ at ζ = 0.0, but their general variation trends are similar. Therefore, it can be seen that a better prediction not only requires an appropriate GB atom selection method, but an appropriate value of ζ. In fact, the MREs of Al and Ni are also dependent on ζ, as seen from Figures S15 and S20 in Supplementary Material.

## 4. RNN-Based Predictions

**Figure 4:**
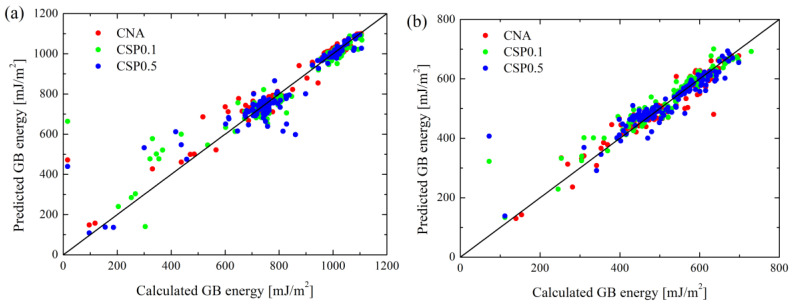

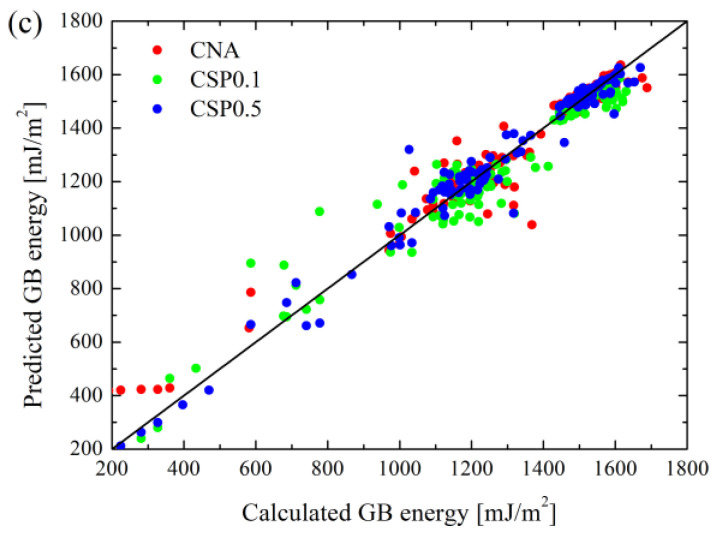
A comparison of RNN-based predictions and MD results for (**a**) Cu, (**b**) Al and (**c**) Ni. Note that predictions are made by considering the parameter ζ corresponding to the minimum MAE of three GB atom selection methods, as shown in Figure 5.

In this section, we discuss the predictions using the RNN method. Due to the dimension reduction in the PCA method, some data is lost. Moreover, a better prediction can be achieved provided that GB types are distinguished based on the tilt axis; i.e., <100> and <110>. In comparison to PCA, on the other hand, the RNN method is highly nonlinear. Considering all of these factors, we do not distinguish GB types for each metal; i.e., the prediction is performed using the full data for each metal. For each metal, 10-fold cross validation is performed, with each fit being performed on a training set consisting of 70% of the total training set selected at random, with the remaining 30% used as a holdout set for testing. This yields good convergence of MAE, as shown in Figure S21 in Supplementary Materials. Figure 4 shows the prediction results of the RNN method considering three GB atom selection methods. A preliminary comparison between PCA and RNN, as shown in Figure 2 and Figure 4, reveals that the RNN method gives a better prediction. This is not surprising due to the higher nonlinearity of the RNN method.

**Figure 5:**
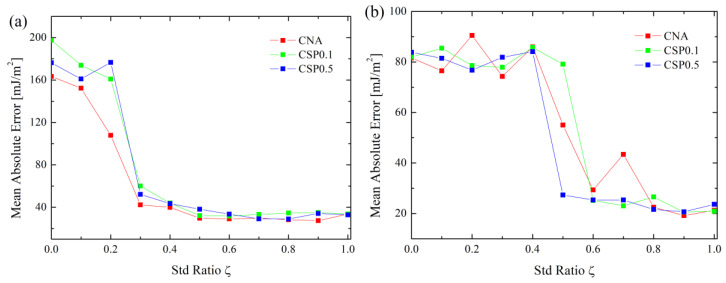

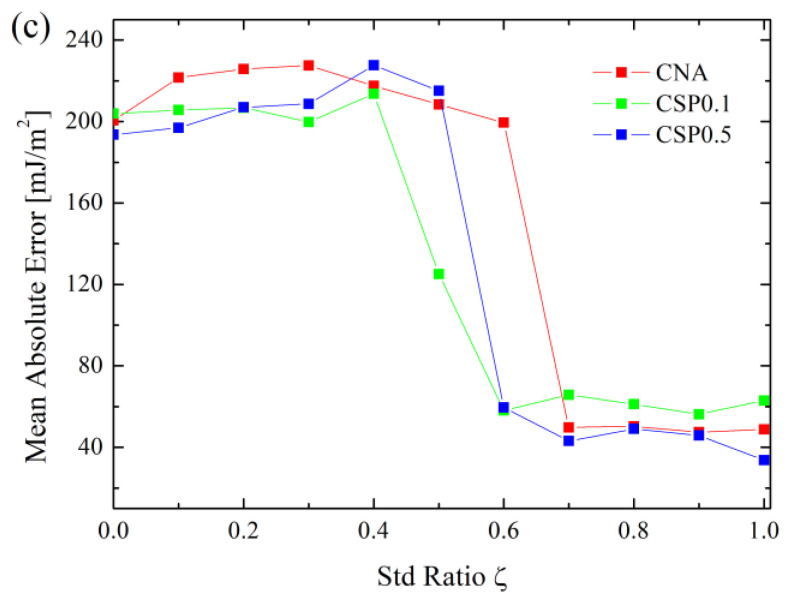
MAE of RNN prediction vs. ζ of three GB atom selection methods for (**a**) Cu, (**b**) Al and (**c**) Ni.

We further evaluated the MAE of RNN predictions for three GB atom selection methods and three metals, as shown in Figure 5. Clearly, with increasing ζ, there is a sudden drop in the MAE. Meanwhile, such a drop in CNA, CSP0.1 and CSP0.5 for the same metal almost occurs at the same value of ζ. Moreover, MAEs for Cu, Al and Ni suddenly drop by ~75%, ~75% and ~70% at ζ_crit_ ≈ 0.3, 0.6 and 0.7. After the sudden drops, all curves approach plateaus with nearly the same MAE, regardless of the three GB atom selection methods. This evidences the significant dependence of ζ in RNN prediction, and also implies that considerable errors will be caused in RNN prediction when letting ζ = 0. To avoid such errors, the standard deviation of PCFs (PCF_std_) must be incorporated into the descriptor. Moreover, the ζ_crit_ of the three metals differ, which implies that the prediction accuracy can be enhanced only when more data scatter information of PCF is considered. ζ_crit_ of Cu is the smallest, while that of Ni is the largest. It suggests that data scatter of the average PCF for Cu is the lowest, but that of Ni is the highest.

## 5. Discussions

**Figure 6:**
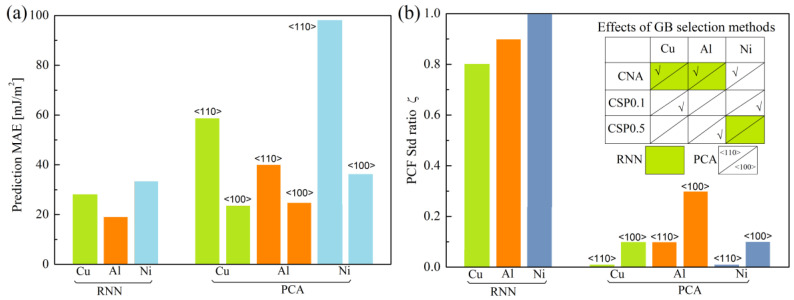
(**a**) Lowest MAE and (**b**) the variation of PCF standard deviation ratio ζ at the lowest MAE for PCA and RNN predictions. Inset in figure (**b**) shows which GB selection methods yield the lowest MAEs for PCA and RNN predictions.

In this study, we predicted GB energies by using PCA-based linear regression and RNN. Figure 6 compares the GB energy prediction properties of two ML methods. From Figure 6a, RNN gives a better prediction than the PCA method. For PCA-based linear regression, linear fitting parameters of GB energy are obtained by dividing a full dataset into two separate datasets according to GBs of <100> and <110> tilt axis for three metals. In doing so, we attempted to weaken the effects of the mutual interference due to <100> and <110> GBs when obtaining linear fitting parameters. Indeed, such a way of treating a dataset for PCA-based predictions decreases the prediction errors (Figure 2). We also tried to further obtain the linear fitting parameters by expressing them as cubic polynomial interpolation functions of an inclination angle of GB *ϕ*, instead of obtaining them from data training. The purpose of doing so was to obtain an empirical GB energy prediction function and intensify the interpretability of ML prediction, which may be impossible for the RNN method. Such a method may work for PCA-based predictions, as evidenced in Figure 2.

An appropriate GB structure descriptor is vital in ML-based GB energy prediction, as whether the descriptor contains the essential information of GB structures or not determines the prediction accuracy. In fact, PCF as a GB structure descriptor [16], compared with those of polyhedral units [34,35], is much easier use. However, the definition of PCF shows that the GB structure is described by an average function. It is believed that the GB structure may not be well described without considering the higher order moment of RDF data from the statistical point of view. Motivated by this, we further incorporated the standard deviation of PCF into the PCF function to further extend the descriptor of the GB structure (Equation (2)). Figure 6b shows that predictions using PCA and RNN are significantly dependent on the parameter and GB atom selection methods for three metals, also suggesting the necessity of considering PCF_std_ in GB structure descriptors.

## 6. Conclusions

In this paper, we studied the GB energies prediction properties of Cu, Al and Ni using two ML methods; i.e., PCA-based linear regression and RNN. We considered the asymmetric GBs Σ3, Σ5, Σ9, Σ11, Σ13 and Σ17 of <110> and <100> types. Atomistic models were constructed and relaxed using the MD method. By extending the PCF-based GB structure descriptor and using three methods of selecting GB atoms, we compared the prediction of two ML methods. The main conclusions of this study were drawn as:

For the three metals, the lowest MAE can be obtained when ζ is greater than 0.8 for RNN, while that should be smaller than 0.3 for PCA-based linear regression. This indicates the dependence of GB descriptors on the ML method. Meanwhile, PCF as an average function and GB structure descriptor needs to consider the PCF_std_, by which ML prediction accuracy can be improved. The GB structure descriptor in the form of average structure representation (ASR) may need to further take into account the standard deviation of ASR.

In comparison to RNN, it is indeed possible to intensify or realize the interpretability of ML prediction by using the PCF-based linear regression method, though how to generalize the fitting method of the linear regression function when considering a dataset of different GB misorientations still needs to be addressed.

For a specific ML method, the MAE of the prediction is determined by multiple factors, such as the GB atom selection method and a portion of PCF standard deviation. A better quantitative descriptor of GB structure is a trade-off between computation cost and complexity. It is expected that prediction accuracy can be enhanced by combining those comprehensive descriptors together. Moreover, it will be interesting to examine the performance of GB structure descriptors if we consider GBs of mixed types.

## Figures and Tables

**Table 1:** MAE and MRE of Cu GB energy predictions. Note that this table lists the prediction for PCF*_comb_* computed for CNA-based GB atom selection and ζ is taken as 0.5.

Error	∑3	∑9	∑11	∑5	∑13	∑17
MAE (mJ/m^2^)	99.72	50.86	70.48	31.70	34.22	64.19
MRE (%)	42.54	6.40	11.89	3.14	3.48	6.30

## Data Availability

The data that supports the findings of this study are available in the supplementary material of this article.

## Supplementary Materials

The following supporting information can be downloaded at: https://www.mdpi.com/article/10.3390/ma16031197/s1, Figure S1. Schematic of the 3D bicrystal GB model. Figure S2. Three methods of selecting GB at-oms in a Σ3 Cu GB. Red atoms in the center of model are those selected out of whole model based on different methods. Figure S3. PCF(r) and PCFcomb(r) of GBs ∑5(310), ∑9(114) and ∑3(111) in Al and Ni. Figure S4. the explained variance percentage for first-ten principle components by considering full data set of Cu. Figure S5. PCA based prediction results of Cu <110> GBs using full data. Regression is performed using PCi based on dimensionality reduction and further interpolation as a function of ϕ, respectively. Figure S6. PCA based prediction results of Cu <100> GBs using full data. Regression is performed using PCi based on dimensionality reduction and further interpolation as a function of ϕ, respectively. Figure S7. PCA based prediction results of Cu <110> GBs using partitioned <110> data. Regression is performed using PCi based on dimensionality reduction and further interpolation as a function of ϕ, respectively. Figure S8. PCA based prediction results of Cu <100> GBs using portioned <100> data. Regression is performed using PCi based on dimensionality reduction and further interpolation as a function of ϕ, respectively. Figure S9. Comparison of Computed PCi with cubic interpolated PCi as a function of inclination angle for <110> type Cu GBs. Figure S10. Comparison of Computed PCi with cubic interpolated PCi as a function of inclination angle for <100> type Cu GBs. Figure S11. PCA based prediction results of Al <110> GBs using partitioned <110> data. Regression is performed using PCi based on dimensionality reduction and further interpolation as a function of ϕ, respectively. Figure S12. PCA based prediction results of Al <100> GBs using portioned <100> data. Regression is performed using PCi based on dimensionality reduction and further interpolation as a function of ϕ, respectively. Figure S13. Comparison of Computed PCi with cubic interpolated PCi as a function of inclination angle for <110> type Al GBs. Figure S14. Comparison of Computed PCi with cubic interpolated PCi as a function of inclination angle for <100> type Al GBs. Figure S15. MRE of Al GBs for three methods of selecting GB atoms. Figure S16. PCA based prediction results of Ni <110> GBs using partitioned <110> data. Regression is performed using PCi based on dimensionality reduction and further interpolation as a function of ϕ, respectively. Figure S17. PCA based prediction results of Ni <100> GBs using portioned <100> data. Regression is performed using PCi based on dimensionality reduction and further interpolation as a function of ϕ, respectively. Figure S18. Comparison of Computed PCi with cubic interpolated PCi as a function of inclination angle for <100> type Ni GBs. Figure S19. Comparison of computed PCi with cubic interpolated PCi as a function of inclination angle for <100> type Ni GBs. Figure S20. MRE of Cu GBs for three methods of selecting GB atoms. Figure S21. MAE convergence curve for RNN prediction of Cu GBs. Table S1. Some additional information of all GB models. Table S2. Variation range of GB energies for all GBs of each metal (mJ/m^2^). Table S3. Some material constants and parameter used for computing PCF. Table S4. Cubic interpolation of PCi as a function of inclination angle for all Cu GBs. Table S5. Cubic interpolation of PCi as a function of inclination angle for all Al GBs. Table S6. Cubic interpolation of PCi as a function of inclination angle for all Ni GBs.
