# Supplementary material for: Standard Deviation Effect of Average Structure Descriptor on Grain Boundary Energy Prediction
Source: Materials (Basel). 2023 Jan 31;16(3):1197. doi: 10.3390/ma16031197 (PMC9919491; doi:10.3390/ma16031197)
Supplement: Supplementary file 1 [file materials-16-01197-s001.zip › materials-2113970-supplementary.pdf]

materials MDPI

Supplementary Material

Standard Deviation Effect of Average Structure Descriptor on Grain Boundary Energy Prediction

Ruoqi Dang and Wenshan Yu *

State Key Laboratory for Strength and Vibration of Mechanical Structures, Shaanxi Engineering Laboratory for Vibration Control of Aerospace Structures, School of Aerospace Engineering, Xi'an Jiaotong University, Xi'an 710049, China
* Correspondence: wenshan@xjtu.edu.cn

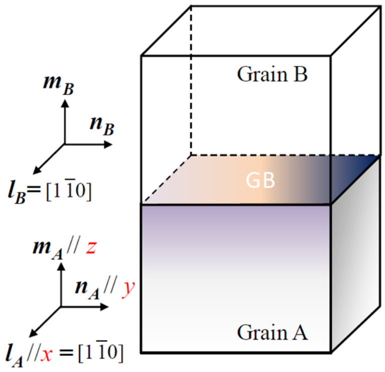

Figure S1. Schematic of the 3D bicrystal GB model.

The GB energy γ_{GB} is computed as the total excess potential energies ($\sum_i (E^i - E^{Coh})$) of atoms in the slab centered on the GB divided by GB area A_{GB}, i.e.,

$$\gamma_{GB} = \sum_i (E^i - E^{Coh}) / A_{GB} \quad \text{S1}$$

where E^{Coh} is the cohesive energy of each metal (see Table S8). E^i is potential energy of each atom in the slab. The slab thickness containing GB is taken as 60 Å. In this way, not only are the effects of free surfaces excluded, but also are imperfect effects due to GB included.

Table S1. Some additional information of all GB models.

	Tilt axis	Inclination angle	Number of models	Variation range of number of atoms
∑3	[110]	0°~90°	91	1152~215,424
∑5	[100]	0°~45°	108	3200~295,920
∑9	[110]	0°~90°	70	3456~544,848
∑11	[110]	0°~90°	66	4224~722,240
∑13	[100]	0°~45°	92	4160~769,521
∑17	[100]	0°~45°	37	2168~271,968

Table S2. Variation range of GB energies for all GBs of each metal (mJ/m²).

	Cu	Al	Ni
Σ3	16~796	72~653	69~1438
Σ5	898~1118	491~762	1343~1624
Σ9	661~944	444~635	970~1521
Σ11	303~812	147~536	469~1379
Σ13	783~1050	429~663	1159~1603
Σ17	850~1107	551~691	1495~1880

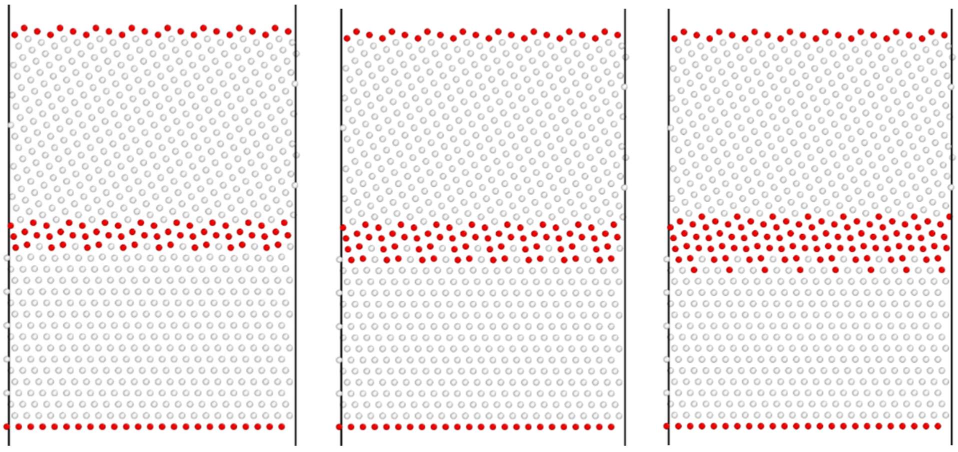

（a）CNA （b）CSP>0.5 （c）CSP>0.1

Figure S2. Three methods of selecting GB atoms in a Σ3 Cu GB. Red atoms in the center of model are those selected out of whole model based on different methods.

Table S3. Some material constants and parameter used for computing PCF.

	Cu	Al	Ni
E_{coh} (eV/atom)	3.45013	3.35994	4.45
Lattice constant a (Å)	3.615	4.05	3.52
Cutoff radius r_{cutoff} (Å)	5.5	6.3	6.725
Atom density n_0 (Å^{-3})	$8.467*10^{-2}$	$6.02*10^{-2}$	$9.17*10^{-2}$
Bandwidth h^e (Å)	0.38	0.42	0.37

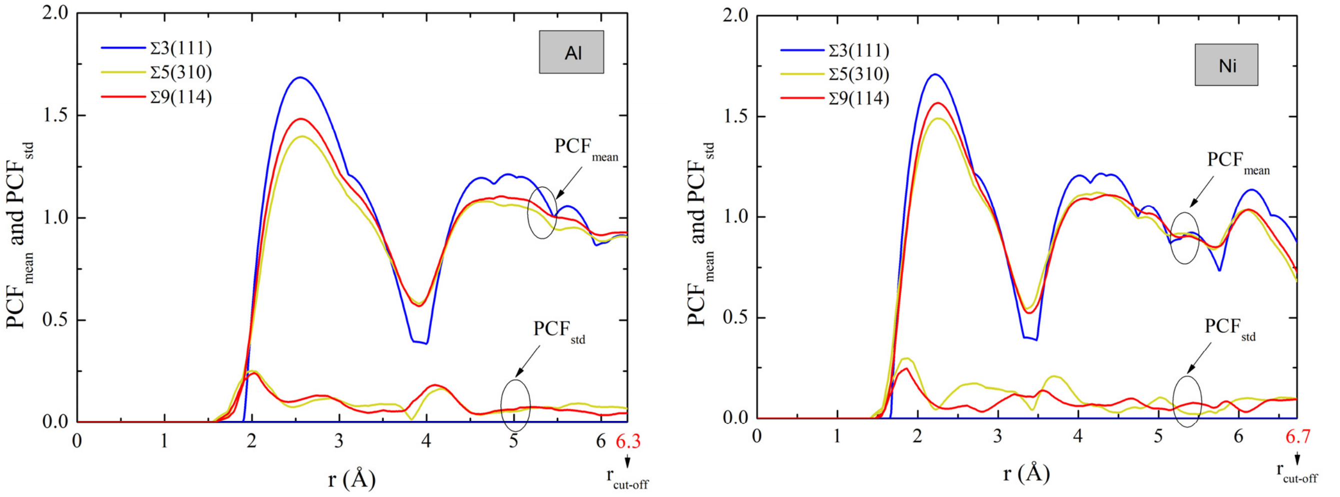

Figure S3. PCF(r) and PCF_{comb}(r) of GBs Σ5(310), Σ9(114) and Σ3(111) in Al and Ni.

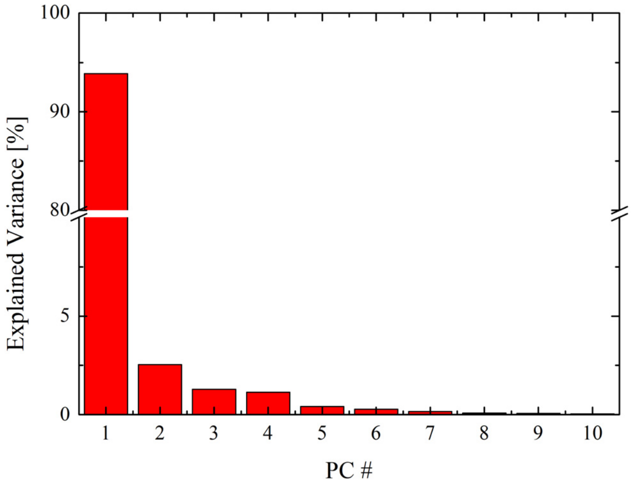

Figure S4. the explained variance percentage for first-ten principle components by considering full data set of Cu.

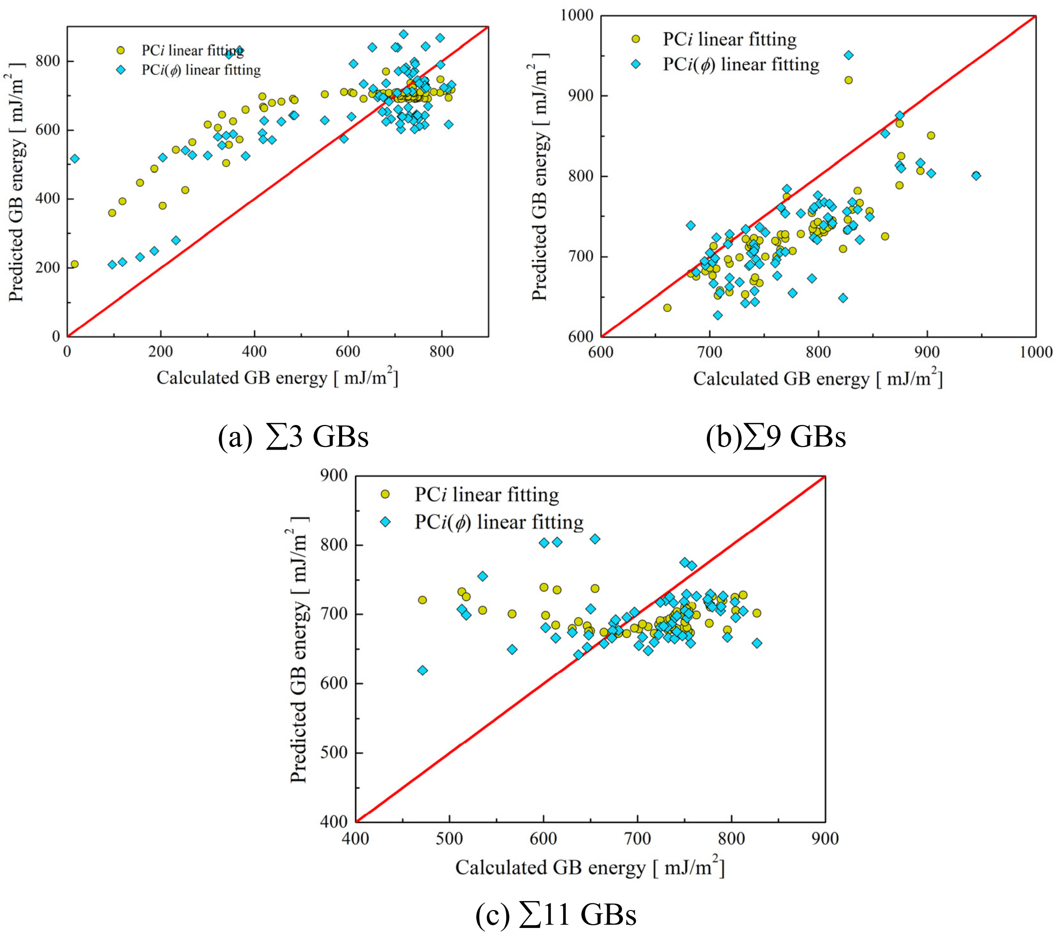

Figure S5. PCA based prediction results of Cu <110> GBs using *full data*. Regression is performed using PC_i based on dimensionality reduction and further interpolation as a function of ϕ, respectively.

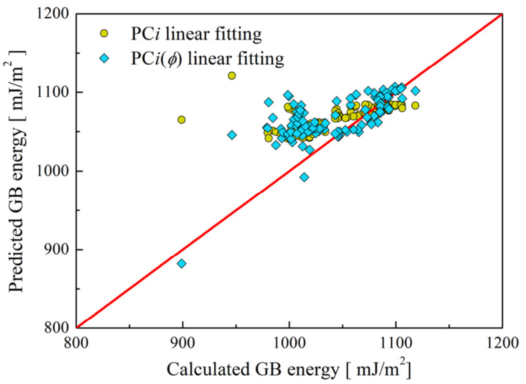

(a) ∑5 GBs

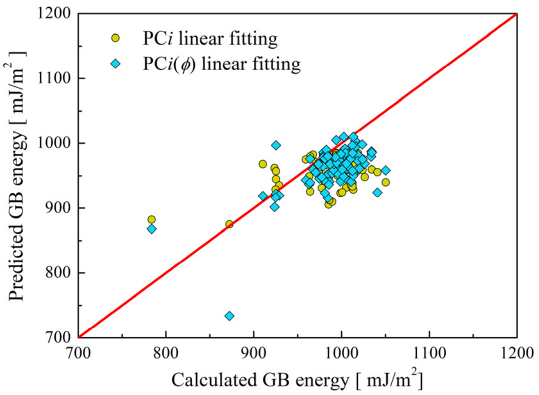

(b)∑13 GBs

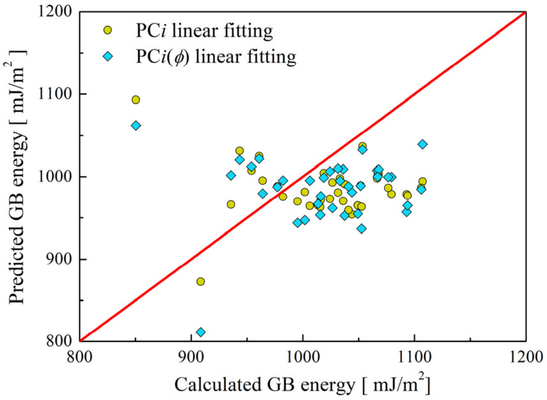

(c)∑17 GBs

Figure S6. PCA based prediction results of Cu <100> GBs using *full data*. Regression is performed using PC$_i$ based on dimensionality reduction and further interpolation as a function of ϕ, respectively.

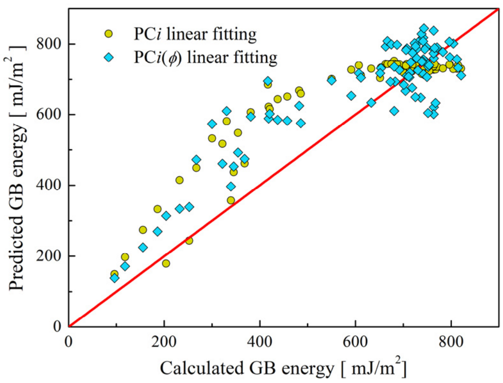

(a) Σ3 GBs

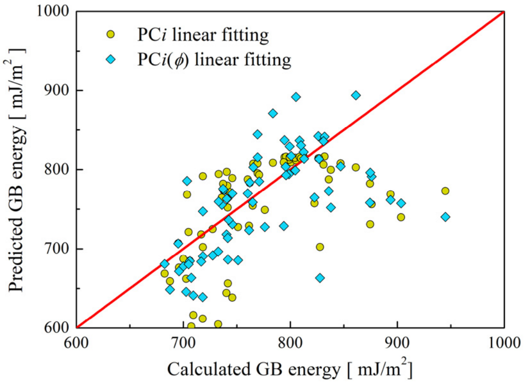

(b)Σ9 GBs

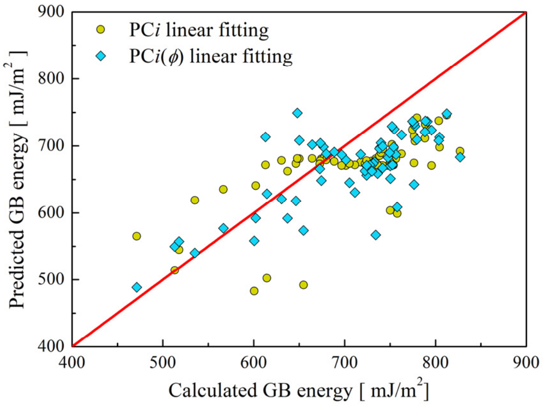

(c)∑11 GBs

Figure S7. PCA based prediction results of Cu <110> GBs using *partitioned <110> data*. Regression is performed using PC$_i$ based on dimensionality reduction and further interpolation as a function of ϕ, respectively.

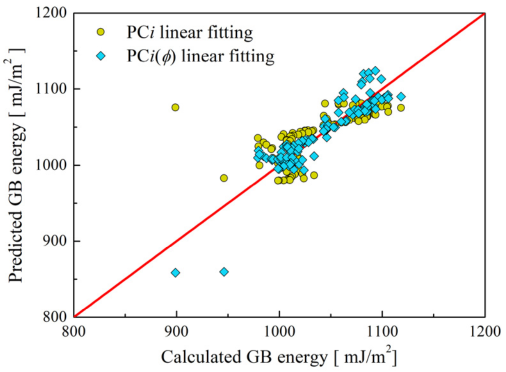

(a)∑5 GBs

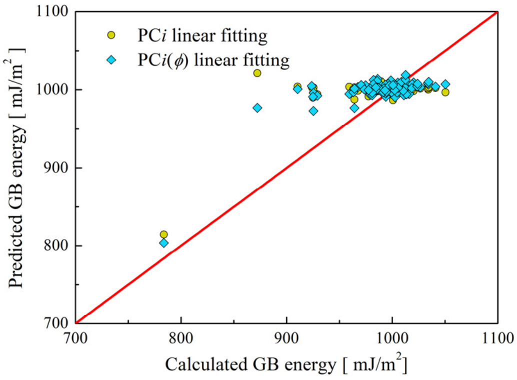

(b)∑13 GBs

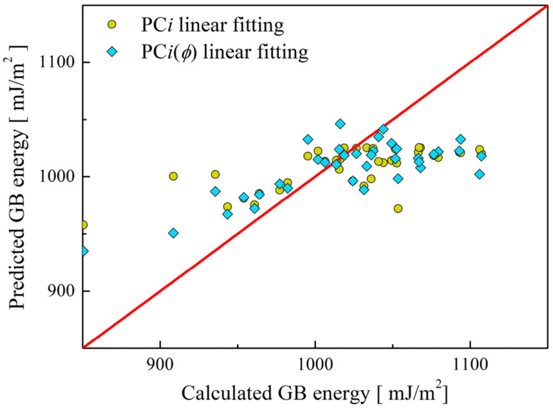

(c)∑17 GBs

Figure S8. PCA based prediction results of Cu <100> GBs using *portioned <100> data*. Regression is performed using PC$_i$ based on dimensionality reduction and further interpolation as a function of ϕ, respectively.

Linear regressions of <110> and <100> type of Cu GBs using three PC$_i$:

$$\hat{\gamma}_{GB[110]} = -620.98PC_1 - 762.0PC_2 - 662.24PC_3 + 668.76 \quad \text{S2}$$

$$\hat{\gamma}_{i[100]} = -60.16PC_1 - 368.34PC_2 - 383.27PC_3 + 1017.73 \quad \text{S3}$$

Table S4. Cubic interpolation of PC_i as a function of inclination angle for all Cu GBs.

$$PC_i = A\phi^3 + B\phi^2 + C\phi + D$$

	PC #	<110>			<100>		
		Σ3	Σ9	Σ11	Σ5	Σ13	Σ17
**A**	1	2.80e-7	-1.76e-6	-3.09e-6	5.02e-6	3.95e-5	-1.03e-4
	2	3.37e-6	-2.19e-6	-7.84e-7	7.13e-6	3.29e-6	6.28e-6
	3	4.77e-7	3.38e-6	-2.33e-7	1.83e-5	-4.47e-6	-1.51e-6
B	1	2.30e-4	1.80e-4	3.90e-4	3.91e-4	-8.99e-4	7.86e-3
	2	-4.70e-4	3.75e-4	1.30e-4	-4.02e-4	1.93e-5	-3.92e-4
	3	-1.09e-5	-3.59e-4	9.83e-5	-1.06e-3	4.60e-4	1.42e-4
C	1	-1.8e-2	-4.16e-3	-1.26e-2	-2.31e-2	-1.25e-2	-0.18
	2	2.00e-2	-1.90e-2	-6.28e-3	9.70e-3	-1.44e-3	9.75e-3
	3	3.17e-4	8.08e-3	-1.09e-2	1.19e-2	-1.03e-2	-3.70e-3
D	1	0.20	-3.90e-2	0.12	0.23	0.15	1.06
	2	-0.18	0.17	6.59e-2	-0.17	6.91e-2	-6.13e-2
	3	-7.50e-2	2.72e-2	0.30	2.11e-2	5.82e-2	2.07e-2

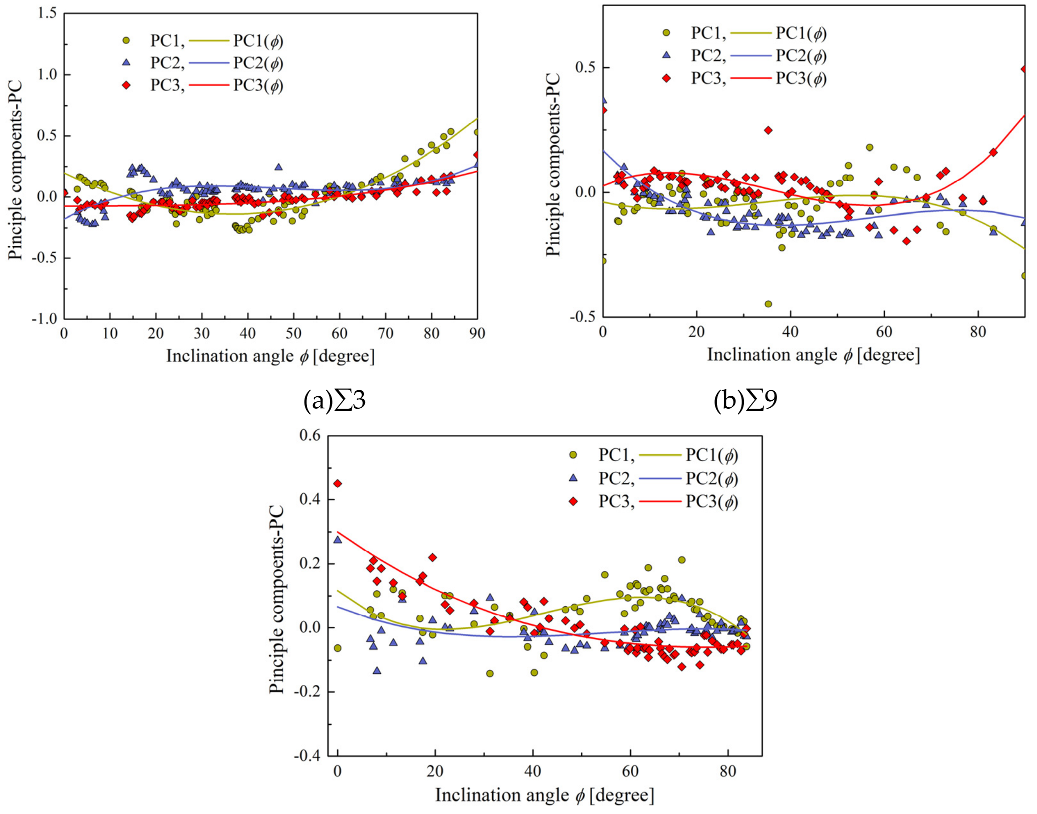

(a)Σ3 (b)Σ9

(c)Σ11

Figure S9. Comparison of Computed PC_i with cubic interpolated PC_i as a function of inclination angle for <110> type Cu GBs.

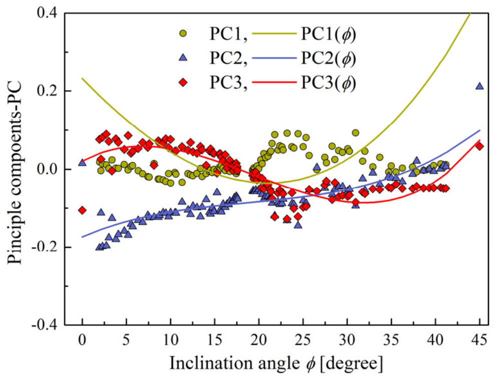

(a)∑5

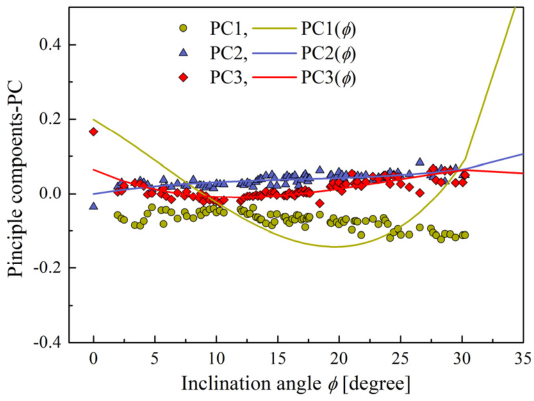

(b)∑13

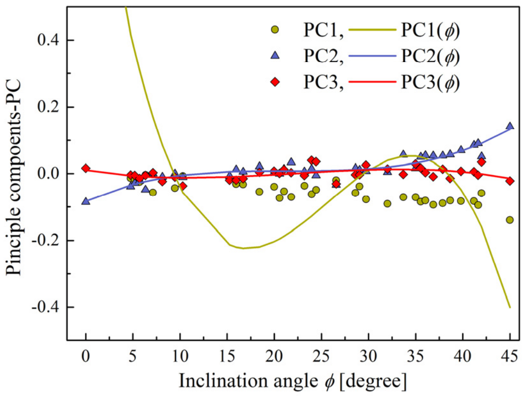

(c)∑17

Figure S10. Comparison of Computed PC$_i$ with cubic interpolated PC$_i$ as a function of inclination angle for <100> type Cu GBs.

In the following, we only shown the PCA prediction results of Al and Ni based on <110> and <100> partitioned data.

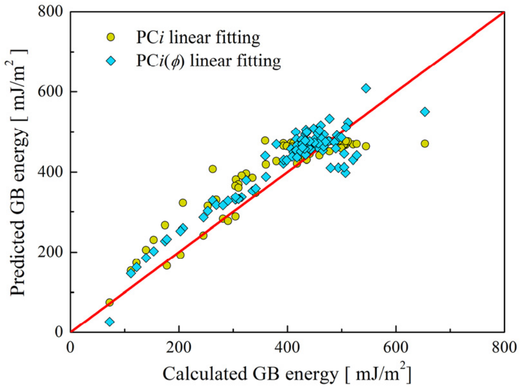

(a)∑3

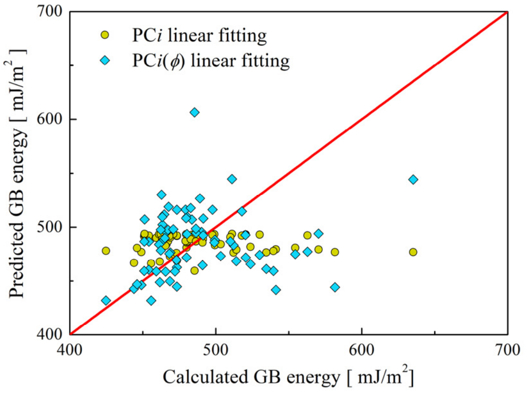

(b)∑9

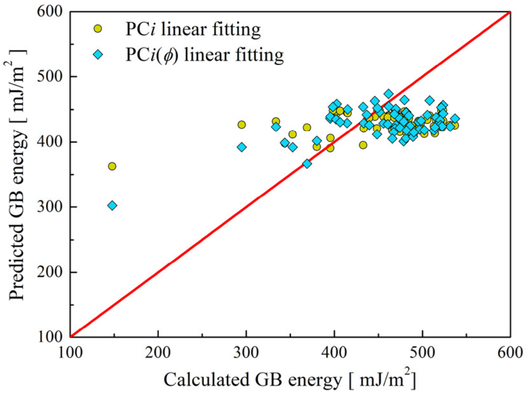

(c)Σ11

Figure S11. PCA based prediction results of Al <110> GBs using *partitioned <110> data*. Regression is performed using PC$_i$ based on dimensionality reduction and further interpolation as a function of ϕ, respectively.

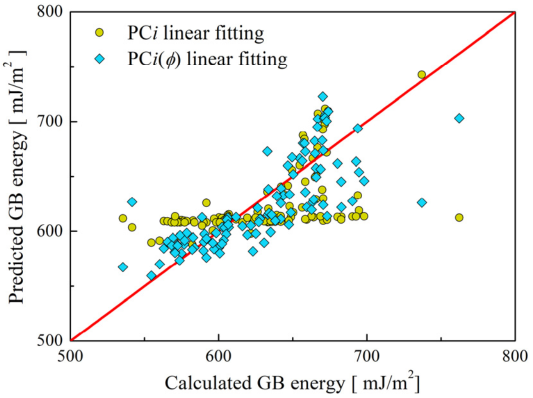

(a)Σ5

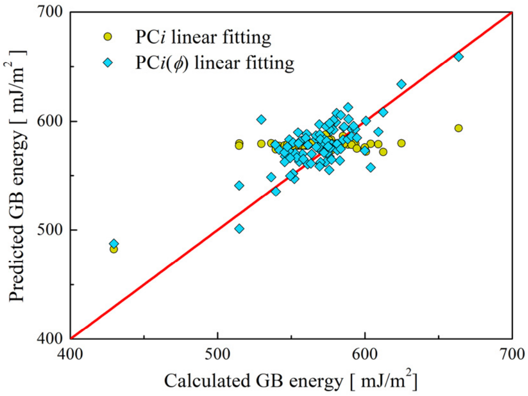

(b)Σ13

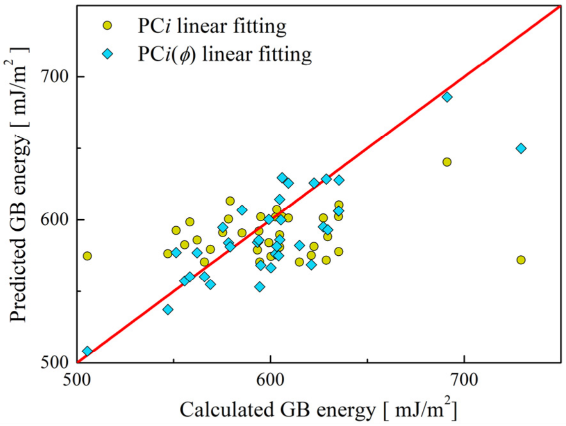

(c)Σ17

Figure S12. PCA based prediction results of Al <100> GBs using *portioned <100> data*. Regression is performed using PC$_i$ based on dimensionality reduction and further interpolation as a function of ϕ, respectively.

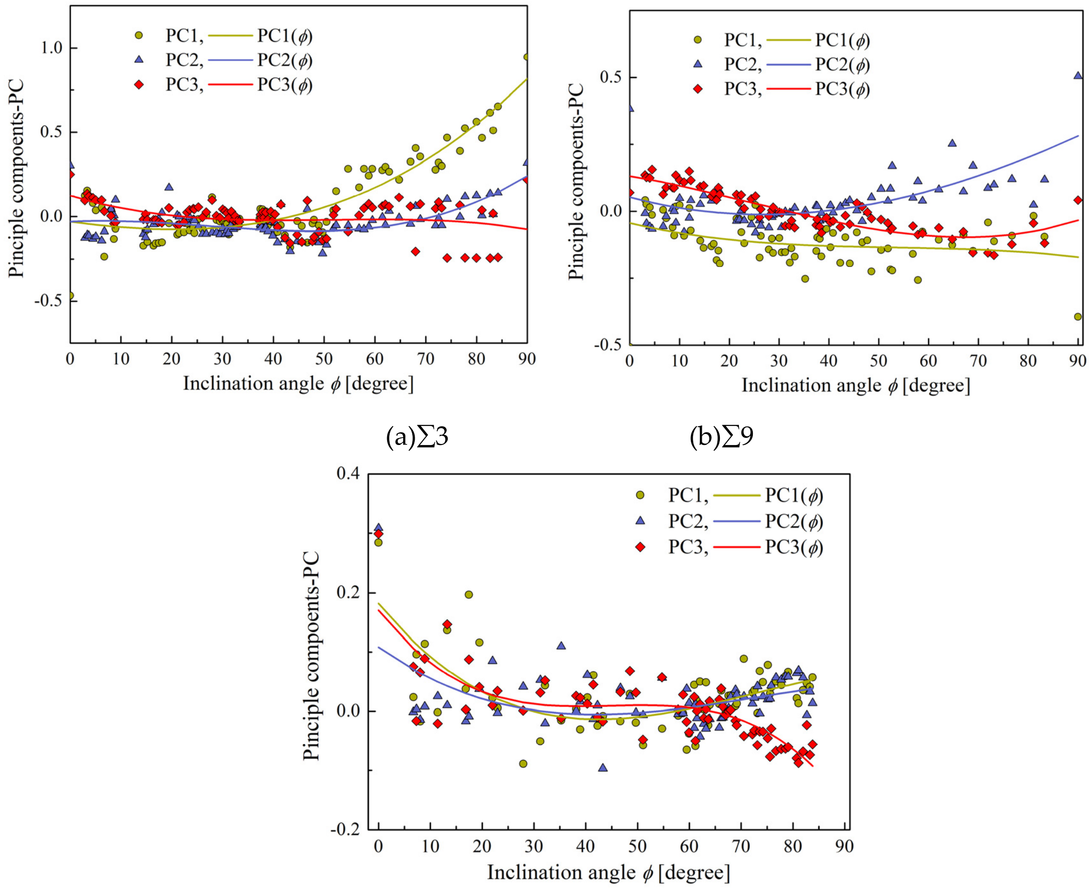

(a)∑3 (b)∑9

(c)∑11

Figure S13. Comparison of Computed PCi with cubic interpolated PCi as a function of inclination angle for <110> type Al GBs.

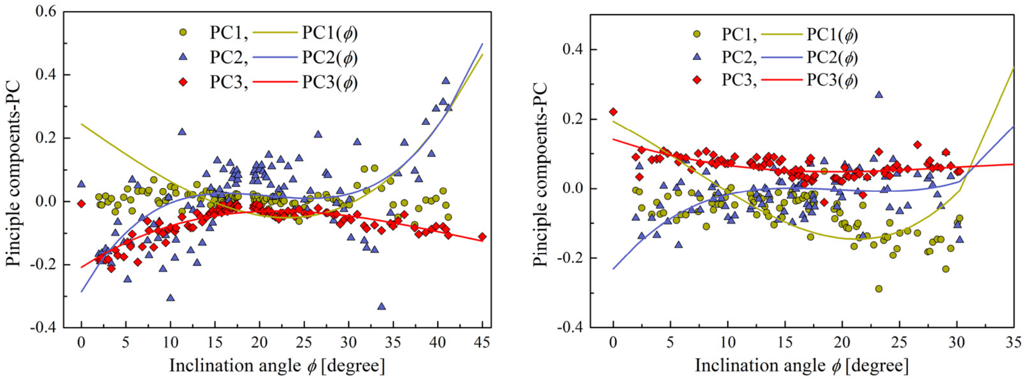

(a)∑5 (b)∑13

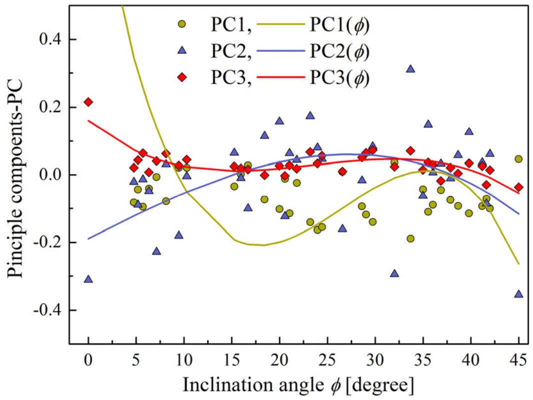

(c)∑17

Figure S14. Comparison of Computed PC_i with cubic interpolated PC_i as a function of inclination angle for <100> type Al GBs.

Linear regressions of <110> and <100> type of Al GBs using three PC_i :

$$\hat{\gamma}_{i[110]} = -409.24PC_1 - 120.89PC_2 - 45.44PC_3 + 442.35 \quad \text{S5}$$

$$\hat{\gamma}_{i[100]} = -16.95PC_1 - 197.40PC_2 - 393.12PC_3 + 600.40 \quad \text{S6}$$

Table S5. Cubic interpolation of PC_i as a function of inclination angle for all Al GBs.

	$PC_i = A\phi^3 + B\phi^2 + C\phi + D$						
	PC #	<110>			<100>		
		∑3	∑9	∑11	∑5	∑13	∑17
**A**	1	8.52e-7	-4.80e-7	-8.27e-7	1.16e-5	3.23e-5	-8.23e-5
	2	1.74e-6	-2.04e-7	-4.73e-7	3.89e-5	3.45e-5	-5.08e-6
	3	-1.93e-6	8.17e-7	-1.88e-6	4.20e-6	-4.15e-6	-1.57e-5
B	1	7.25e-5	7.70e-5	1.79e-4	1.80e-5	-6.23e-4	6.56e-3
	2	-1.41e-4	1.02e-4	1.04e-4	-2.44e-3	-2.03e-3	-5.85e-5
	3	1.84e-4	-5.99e-5	2.54e-4	-5.53e-4	4.06e-4	1.11e-3
C	1	-4.10e-3	-4.46e-3	-1.07e-2	-1.94e-2	-1.73e-2	-0.16
	2	1.68e-3	-5.03e-3	-6.24e-3	4.86e-2	3.82e-2	1.45e-2
	3	-9.10e-3	-3.06e-3	-1.12e-2	1.82e-2	-1.11e-2	-2.31e-2
D	1	-2.96e-2	-4.32e-2	0.18	0.24	0.19	0.95
	2	-3.03e-2	5.37e-2	0.11	-0.29	-0.23	-0.20
	3	0.12	0.13	0.17	-0.21	0.14	0.16

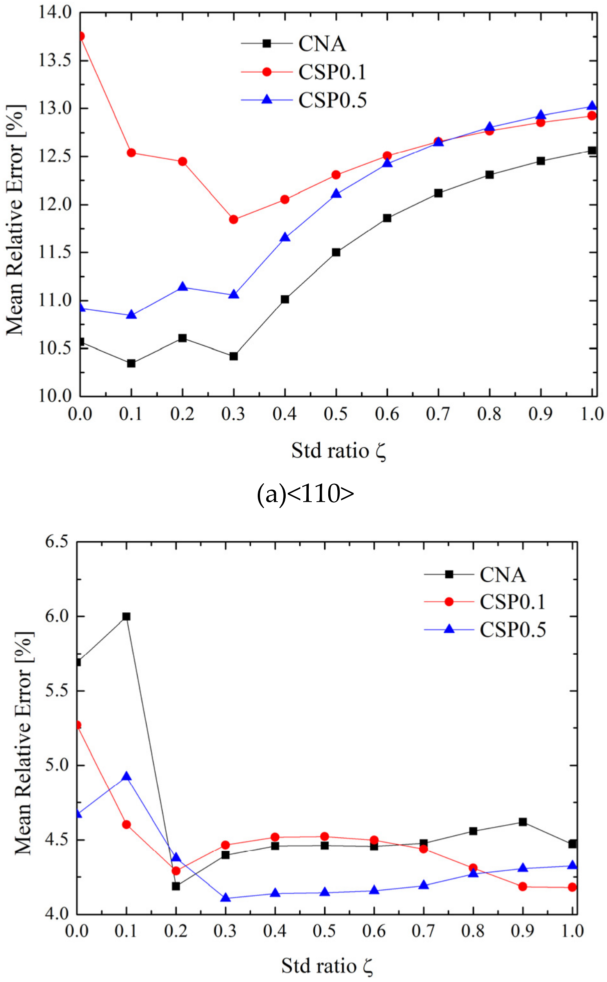

(a)<110>

(b)<100>

Figure S15. MRE of Al GBs for three methods of selecting GB atoms.

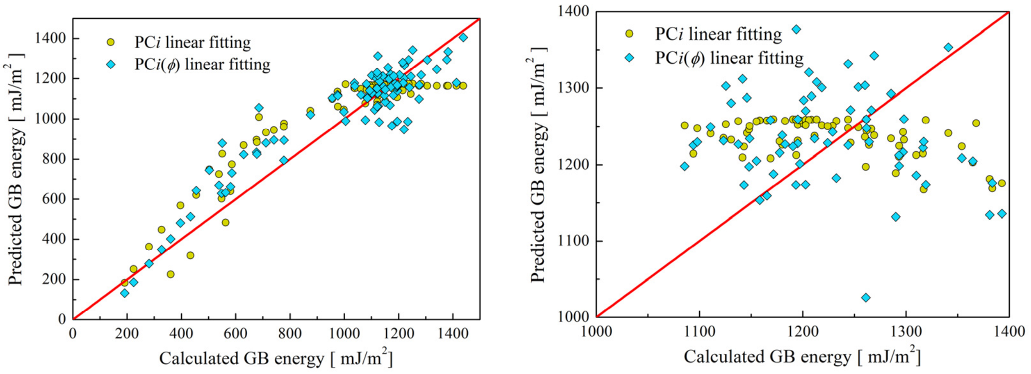

(a)Σ3 (b)Σ9

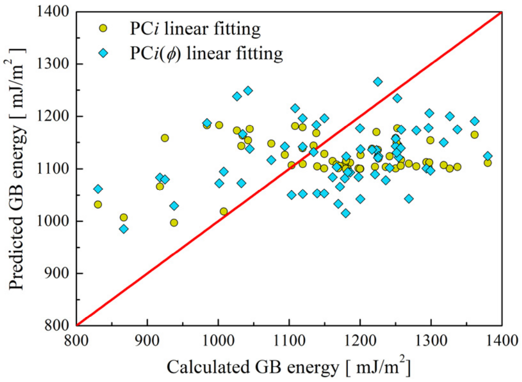

(c)Σ11

Figure S16. PCA based prediction results of Ni <110> GBs using *partitioned <110> data*. Regression is performed using PC$_i$ based on dimensionality reduction and further interpolation as a function of ϕ, respectively.

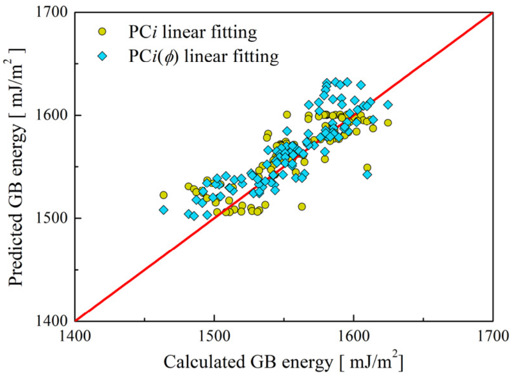

(a)Σ5

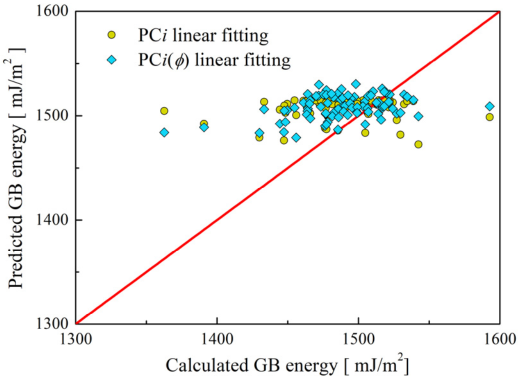

(b)Σ13

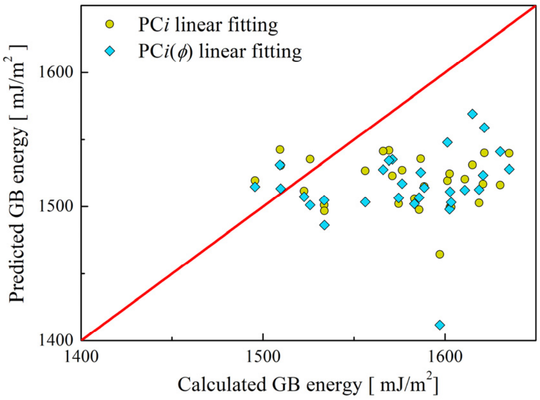

(c)Σ17

Figure S17. PCA based prediction results of Ni <100> GBs using *portioned <100> data*. Regression is performed using PC$_i$ based on dimensionality reduction and further interpolation as a function of ϕ, respectively.

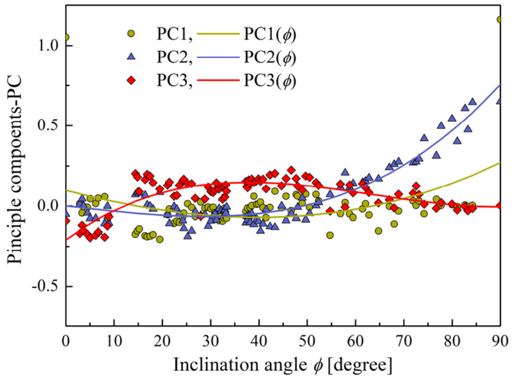

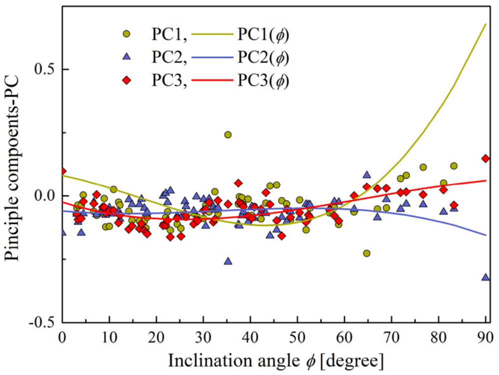

(a)Σ3 (b)Σ9

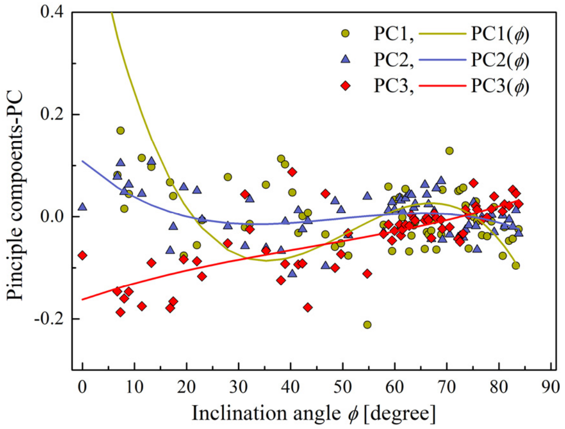

(c)Σ11

Figure S18. Comparison of Computed PC_i with cubic interpolated PC_i as a function of inclination angle for <100> type Ni GBs.

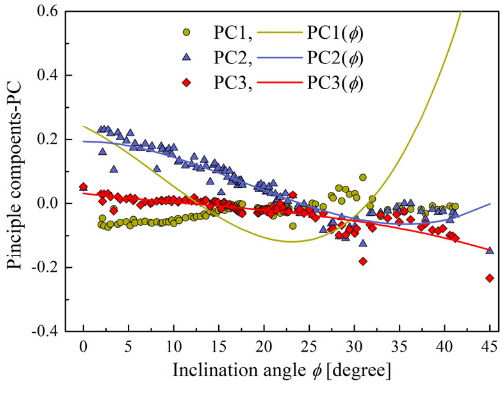

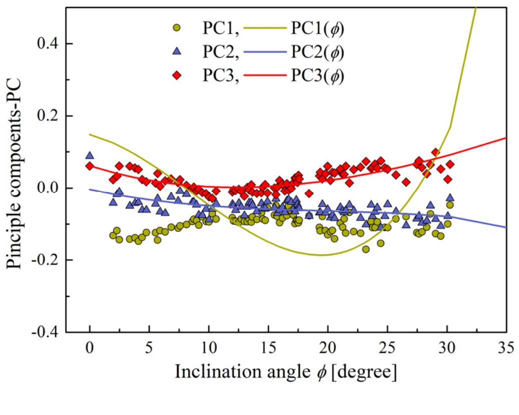

(a)Σ5 (b)Σ13

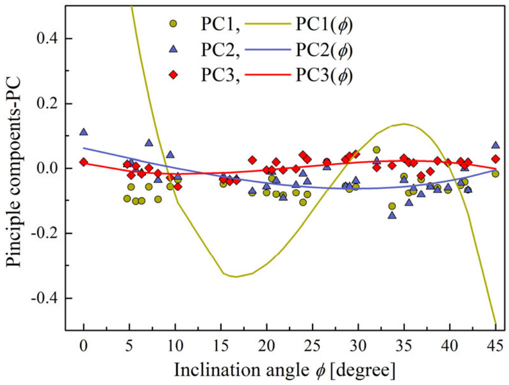

(c)∑17

Figure S19. Comparison of computed PC_i with cubic interpolated PC_i as a function of inclination angle for <100> type Ni GBs.

Linear regressions of <110> and <100> type of Ni GBs using three PC_i :

$$\hat{\gamma}_{i[110]} = -228.66PC_1 - 1522.09PC_2 - 383.09PC_3 + 1111.31 \quad \text{S7}$$

$$\hat{\gamma}_{i[100]} = -55.83PC_1 + 438.28PC_2 - 192.74PC_3 + 1533.02 \quad \text{S8}$$

Table S6. Cubic interpolation of PC_i as a function of inclination angle for all Ni GBs.

	$PC_i = A\phi^3 + B\phi^2 + C\phi + D$						
	PC #	[110]			[100]		
		∑3	∑9	∑11	∑5	∑13	∑17
**A**	1	3.52e-7	2.85e-6	-7.03e-6	3.28e-5	6.90e-5	-1.61e-4
	2	1.53e-6	-7.66e-7	-1.35e-6	1.15e-5	-5.25e-6	2.34e-6
	3	2.17e-6	-7.21e-7	3.03e-6	-1.83e-6	-3.86e-6	-5.67e-6
B	1	7.67e-5	-1.40e-4	1.08e-3	-8.47e-4	-1.79e-3	1.25e-2
	2	-6.69e-6	7.55e-5	2.00e-4	-6.16e-4	3.12e-4	2.38e-6
	3	-4.16e-4	1.39e-4	-3.96e-5	6.65e-5	4.89e-4	3.95e-4
C	1	-7.83e-3	-3.84e-3	-4.99e-2	-1.35e-2	-8.53e-3	-0.28
	2	-3.37e-3	-1.65e-3	-8.86e-3	1.31e-4	-7.13e-3	-6.32e-3
	3	2.21e-2	-5.75e-3	3.51e-3	-3.18e-3	-1.03e-2	-6.65e-3
D	1	9.74e-2	8.15e-2	0.64	0.24	0.15	1.66
	2	7.15e-4	-5.92e-2	0.11	0.19	-3.90e-3	6.14e-2
	3	-0.21	-2.44e-2	-0.16	3.09e-2	6.14e-2	1.55e-2

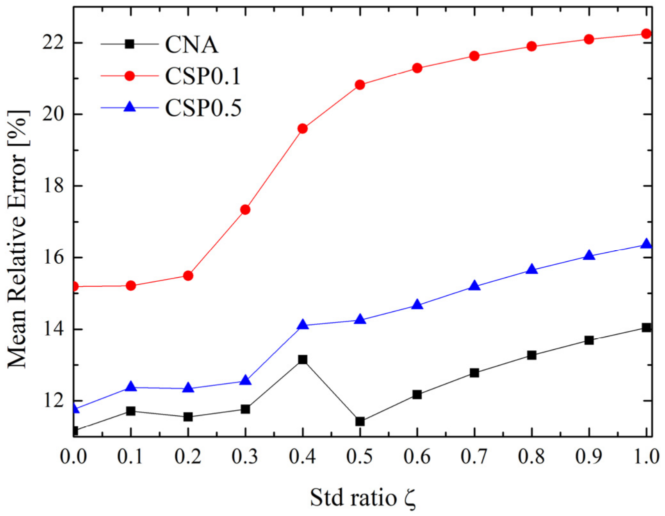

(a) <110>

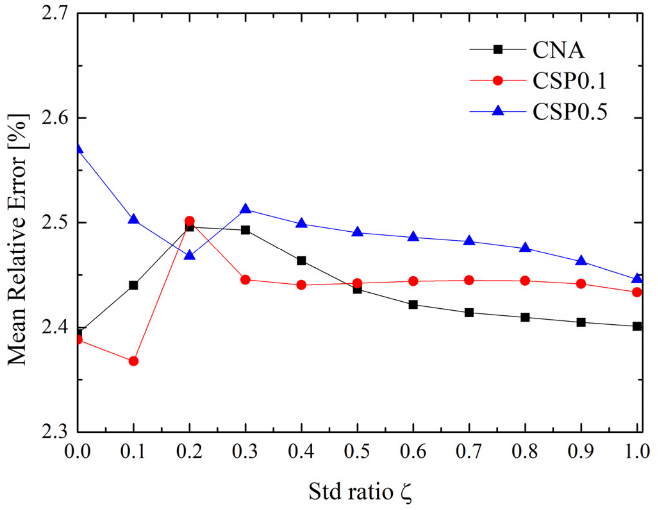

(b) <100>

Figure S20. MRE of Cu GBs for three methods of selecting GB atoms.

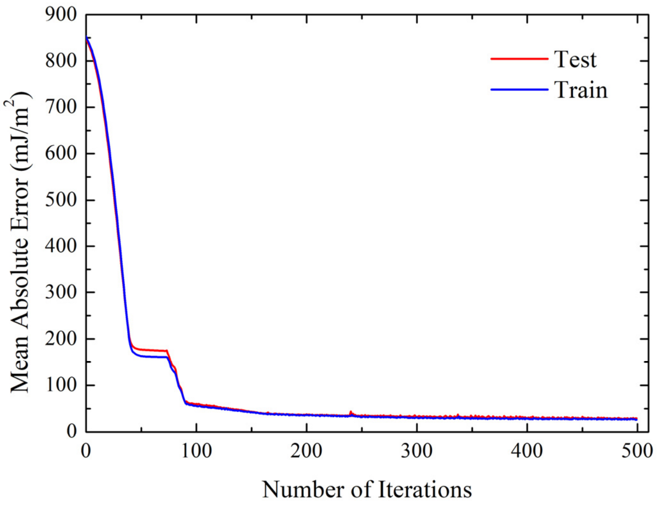

Figure S21. MAE convergence curve for RNN prediction of Cu GBs.
